# Genome-wide association study identifies a novel locus associated with psychological distress in the Japanese population

**DOI:** 10.1038/s41398-019-0383-z

**Published:** 2019-01-31

**Authors:** Hisatsugu Koshimizu, Shun Nogawa, Shinya Asano, Masashi Ikeda, Nakao Iwata, Shoko Takahashi, Kenji Saito, Tsuyoshi Miyakawa

**Affiliations:** 1 Division of Systems Medical Science, Institute for Comprehensive Medical Science, Fujita Health University, 1-98 Dengakugakubo, Kutsukake-cho, Toyoake, 470-1192 Japan; 2 Genequest Inc., 5-29-11 Shiba, Minato Ward, Tokyo, 108-0014 Japan; 3 Department of Psychiatry, Fujita Health University School of Medicine, 1-98 Dengakugakubo, Kutsukake-cho, Toyoake, 470-1192 Japan

## Abstract

Major depressive disorder (MDD) is a common and disabling psychiatric disorder. A recent mega analysis of genome-wide association studies (GWASs) identified 44 loci associated with MDD, though most of the genetic etiologies of the MDD/psychological distress remain unclear. To further understand the genetic basis of MDD/psychological distress, we conducted a GWAS in East Asia with more than 10,000 participants of Japanese ancestry who had enrolled in a direct-to-consumer genetic test. After quality control on the genotype data, 10,330 subjects with a total of 8,567,708 imputed SNPs were eligible for the analysis. The participants completed a self-administered questionnaire on their past medical history and health conditions that included the 6-item Kessler screening scale (K6 scale) for psychological distress (cut-off point of 5) and past medical history of MDD, resulting in 3981 subjects assigned to “psychologically distressed group” [cases], and the remaining 6349 subjects were assigned to the “non-psychologically distressed group” [controls]. In this GWAS, we found an association with genome-wide significance at rs6073833 (*P* = 7.60 × 10^−9^) in 20q13.12. This is, to the best of our knowledge, the first large-scale GWAS for psychological distress using data from direct-to-consumer (DTC) genetic tests in a population of non-European-ancestry, and the present study thus detected a novel locus significantly associated with psychological distress in the Japanese population.

## Introduction

Major depressive disorder (MDD) is one of the most common psychiatric disorders, with approximately 15% of the lifetime prevalence. Although the etiology underlying MDD remains unclear, based on the etiological studies, MDD is a multifactorial disorder with multiple genetic and environmental factors that have relatively small effects^1^. Based on this evidence, many genetic studies have sought to identify loci that are significantly (*P* < 5.00 × 10^−8^) associated with MDD, but only a small number of loci that met the criteria have been identified, likely because of an under-powered sample size^2–5^.

Very recently, however, the Psychiatric Genomics Consortium (PGC) carried out meta- and mega-analyses (PGC2) of genome-wide genomic data for MDD in which 130,664 MDD cases and 330,470 controls were assessed, identifying 44 MDD-associated loci with genome-wide significance^6^. In the MDD genome-wide association study (GWAS), including the PGC2 MDD GWAS, the majority of the subjects were of European ancestry. Whereas GWASs targeting non-European samples were limited, as there is only one large scale GWAS for MDD in patients of non-European ancestry that was carried out on Han Chinese women by the China, Oxford and Virginia Commonwealth University Experimental Research on Genetic Epidemiology (CONVERGE) consortium^7^. In addition, the sample size being limited, we previously carried out a genome–wide environmental interaction study (Fujita Health University depressive state GWEIS) and identified novel risk loci for psychological distress in the Japanese population^8^. To further assess the susceptibility loci and genes associated with MDD/psychological distress, large and multi-ethnic datasets are essential. In this context, for large GWAS datasets, a web-based data collection is expected to meet these needs^9^.

In the present study, we carried out a GWAS in an East Asian (Japanese) population in which over ten thousand participants filled out web-based questionnaires on their medical/health conditions, and the questionnaires included a case-finding instrument for MDD, the 6-item Kessler screening scale (K6 scale) for psychological distress^10,11^. This GWAS aimed to (1) detect the loci or pathway for the psychological distress/MDD; (2) to assess whether there are overlaps between the loci identified in the present GeneQuest (GQ) psychological distress GWAS and those found in previous GWASs including the CONVERGE MDD study, PGC2 MDD study, the Genetics of Personality Consortium (GPC2) neuroticism study, and Fujita Health University depressive state GWEIS; and (3) to evaluate genetic correlations between the present psychological distress GWAS results and those of the CONVERGE and PGC2 MDD GWASs. To the best of our knowledge, this is the first large-scale GWAS for MDD/psychological distress using data from direct-to-consumer (DTC) genetic tests carried out in a population of non-European-ancestry.

## Materials and methods

### Study cohort

All participants were the customers of Japanese Direct to Consumer (DTC) genetic testing service, HealthData Lab (Yahoo! Japan Corporation, Tokyo, Japan). The customers purchased or were complementarily provided the genetic testing kit via the internet. They read an agreement, signed a consent form, and registered their IDs on the web page. In the consent form, they signed consent forms agreeing to use their data for research. The participants collected their saliva using Oragene®·DNA(OG-500) (DNA Genotek, Ottawa, Canada), a saliva collection kit. They sent the saliva collection kit and signed consent form to the DTC genetic testing service provider, and a few weeks later, the analysis results were provided via the HealthData Lab web site. We also obtained a second form of consent for the usage of their data in this study by offering an opt-out through the web site at this stage. For participant confidentiality, all individuals were irreversibly anonymized. We approached 11,091 subjects and 11,089 subjects (99.98%) did not opt-out.

### Genotyping assay

DNA was extracted from saliva samples according to the manufacturer’s instructions. Degeneration and concentration of the obtained genomic DNA was performed by gel electrophoresis with PicoGreen® (Thermo Fisher Scientific, Waltham, MA), respectively. When the genomic DNA was degraded or in low concentration, we sent a new saliva collection kit to the customer and requested an additional saliva collection.

Genotyping was performed at RIKEN GENESIS. We used two platforms, the Illumina HumanCore-12 Custom BeadChip and HumanCore-24 Custom BeadChip (Illumina, San Diego, CA) platforms. The Illumina HumanCore-12 Custom BeadChip contained 302,072 markers, including about 2000 our selected markers. The Illumina HumanCore-24 Custom BeadChip contained 309,725 markers, including approximately 3000 our selected markers. When the sample call rate (percentage of SNPs successfully genotyped by sample) for ordinal SNPs, those contained in the Illumina HumanCore platform, was under 85 %, we sent a new saliva collection kit to the customer and re-genotyped. For this study, we selected 296,675 SNPs contained in both genotyping platforms for analyses.

### Phenotype measurement

To determine a participant’s depression phenotype, we used questionnaire responses, which were collected on the DTC genetic testing service web page. The K6 scale was used to assess the psychological distress-related phenotype: (1) “During the past month, how often did you feel so sad that nothing could cheer you up?”, (2) “During the past month, how often did you feel hopeless?”, (3) “During the past month, how often did you feel restless or fidgety?”, (4) “During the past month, how often did you feel nervous?”, (5) “During the past month, how often did you feel that everything was an effort?”, and (6) “During the past month, how often did you feel worthless?”; Answers: Never (score: 0), Not very often (score: 1), Some of the time (score: 2), Most of the time (score: 3), All the time (score: 4). A previous study reported that the sensitivity and specificity of the K6 scale for screening psychological distress were 100 and 68.7%, respectively, with a cut-off point of 5 in the Japanese population^12^. Therefore, we used this cut-off line to dichotomize the samples into two groups, “psychologically distressed group (score: 5~24)” and “non-psychologically distressed group (score: 0~4)”, and subjects with past medical history were removed from “non-psychologically distressed group”.

### Quality control

The QC was assessed for each sample at both the individual and SNP levels. At the individual level, subjects with inconsistent sex information between X chromosomal SNP genotypes and the questionnaire, or low call rates (<0.95), were excluded. In addition, kinship was examined with a pairwise identify-by-descent (IBD) estimation. Individual pairs estimated to be kin (*p*(IBD = 0) < 0.05) were excluded. At the SNP level, SNPs with low call rates (<0.95), low Hardy-Weinberg equilibrium exact test *P* values (<0.001), or low minor allele frequencies (MAFs; <0.01) were excluded. These QC procedures were performed with PLINK v1.07. Finally, 10,892 individuals and 229,276 SNPs from 11,091 individuals remained.

### Population stratification

To estimate genetic ancestry, we applied the principal component analysis in EIGENSOFT v6.1.3^2,3^. To identify continental ancestry, we downloaded HapMap phase 3 individual-level genotypes and merged the HapMap^4^ genotypes with our genotypes. We merged our genotypes with the HapMap data from 4 populations, CEU (Utah residents with Northern and Western Europe ancestry from CEPH collection), YRI (Yoruba in Ibadan, Nigeria), JPT (Japanese in Tokyo, Japan), and CHB (Han Chinese in Beijing, China). The eigenvectors were generated by EIGENSOFT, and the top two of eigenvectors were plotted with R-statistics software v3.2.1. To minimize the ancestry biases, 219 subjects with non-Japanese and Ryukyu ancestry were excluded from further analyses.

### Imputation

Haplotype phasing and imputation were performed using SHAPEIT v2^13^ and IMPUTE2 v2.3.2^14^ with a 1000 Genomes reference panel (phase 3)^15^. Only samples that had passed quality control assessment were imputed. After the imputation, SNPs were assessed using an information threshold of 0.3 and a minor allele frequency threshold of 0.01, giving a final total of 8,567,708 SNPs for analysis.

### Genome-wide association analysis (GWAS)

We used logistic regression to test the association between phenotype and allele in each SNP, assuming an additive model. Covariates included age, sex, and the top five eigenvectors. Odds ratio and 95 % confidence intervals were calculated for effect allele. A Manhattan plot and quantile-quantile plot were constructed using the R software package qqman v0.1.2. The significance level was set at *P* < 5.00 × 10^−8^ and the suggestive significance level was set at *P* < 1.00 × 10^−5^, which is the Bonferroni-correlated threshold for the number of independent variants among the HapMap phase 3 genotyped SNPs. All statistical analyses were performed using PLINK v1.07.

### Gene-set enrichment analysis

To identify biological pathways or gene sets associated with psychological distress, we carried out a pathway analysis using meta-analysis gene-set enrichment of variant associations (MAGENTA) (http://broadinstitute.org/mpg/magenta). MAGENTA implements gene-set enrichment analysis on GWAS data by assessing pathway annotations in web-based databases, including the Data bases: The Gene Ontology (GO), Kyoto Encyclopedia of Genes and Genomes (KEGG), Protein Analysis Through Evolutionary Relationships (PANTHER), BioCarta, Reactome, and Ingenuity databases. Since the 75th percentile cut-off demonstrates greater power than 95th percentile cut-off in interpreting complex diseases with high polygenesis, we used the 75th percentile as the cut-off value for our interpretation^16^.

### Risk profile score analyses

Risk profile score (RPS) analyses were carried our as previously described^17^. Briefly, the statistical analysis software package PRSice v1.23 was used. The P threshold (P_T_) for selecting the “risk” SNPs was set sequentially at 0.001, 0.05, 0.1, 0.2, 0.3, 0.4, and 0.5. SNPs were selected if their *P* values were between 0 and the chosen value of P_T_. The variance for the RPS was estimated using Nagelkerke’s *R*^2^ from a logistic regression mode. We set the type I error rate at 0.001, which is suggested as a conservative threshold^18^.

### Genetic correlation analysis

Linkage disequilibrium (LD) score regression analysis was carried out to examine the genetic correlation, as previously described^17^, using pre-computed LD scores for East Asian (Japanese and Chinese) results^19^. To compare Japanese and European results, we used Popcorn (version 0.9.7) and examined the trans population genetic effect correlation (the correlation coefficient for the per-allele SNP effect sizes, ρ_ge_), and the genetic impact correlation (the correlation coefficient for the population-specific allele variance normalized SNP effect sizes, ρ_gi_)^20^.

## Results

Population sociodemographic features are shown in Table 1. To dichotomize the sample set, we used the K6 scale^21^ to screen subjects with psychological distress and set the cut-off at 5 point. Subjects with a score of 5 or more (psychologically distressed group, *N* = 3981) were classified as cases, and those with score of 0–4 and no past medical history of MDD (non-psychologically distressed group, *N* = 6349) were used as controls.

**Table 1:** Cohort demographics for the GeneQuest data sets for psychological distress assessed using K6

		Total	Case	Control
K6 score			5 ~ 24	0 ~ 4
Number of subjects		10,330	3981	6349
Age (Years)	under 30	992	578	414
	30–45	3206	1490	1716
	45–60	3860	1378	2482
	over 60	2272	535	1737
Sex	Male	5333	1805	3528
	Female	4997	2176	2821

**Fig. 1:**
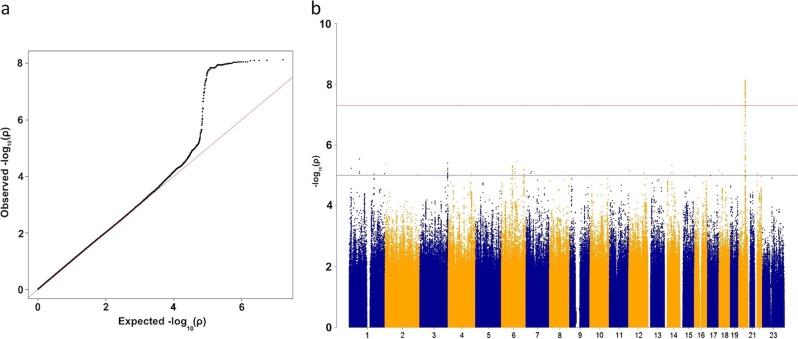
**Association analysis for imputed SNPs**. **a** Quantile-Quantile plot for GeneQuest GWAS for psychological distress assessed using K6. Horizontal and vertical axes indicate expected the *P* values under a null distribution and the observed *P* values respectively. **b** Manhattan Plots show –log10 *P* of the genotyped SNPs. The red line corresponds to *P* = 5.00 × 10^−8^. Gene labels are annotated for the genes closest to the significant SNPs

**Fig. 2:**
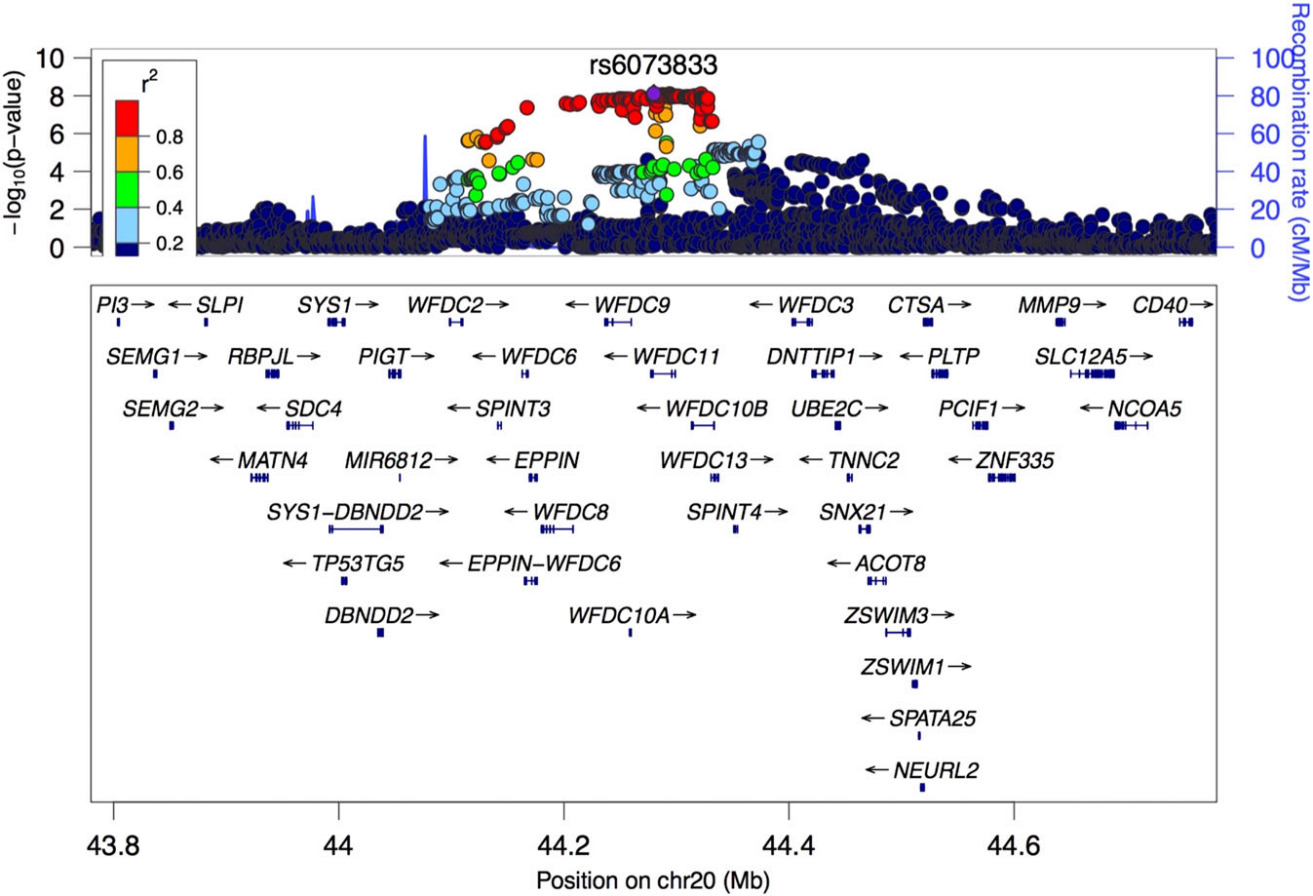
**Regional plot of association signals in the newly identified loci.** The –log10 P of the genotyped SNPs for psychological distress is shown on the left y axis. The recombination rates expressed in centimorgans (cM) per Mb (Megabase) (blue line) are shown on the right y axis. Position in Mb is on the x axis. The most associated SNP is shown as a purple diamond

First, we assessed the loci for the psychological distress/MDD. Quantile–quantile (QQ) and Manhattan plots for psychological distress were assessed with a K6 scale (cut-off point of 5) and are listed in Fig. 1. λgc is 1.020 (95%CI: 1.018–1.022). In this SNP-based association analysis, we detected an association with a genome-wide significant level (*P* < 5.00 × 10^−8^) at rs6073833 (*P* = 7.60 × 10^−9^), a region upstream of *WAP four-disulfide core domain 11* (*WFDC11*) at 20q13.12 (Fig. 2, Table 2). Additional loci with suggestive association levels are shown in Table 2 and Supplementary Table 1. We then assessed the gene-set enrichment for psychological distress, calculated with MAGENTA software^16^. Supplementary Table 3 lists biological pathways and gene sets with a nominal *P*_75% cutoff_ less than 0.01, among which there are biological pathways with a false discovery rate (FDR) less than 0.05, such as platelet-derived growth factor (PDGF) signaling (Nominal *P*_75% cutoff_ = 5.00 × 10^−4^; FDR_75% cutoff_ = 2.72 × 10^−2^) and 14-3-3 signaling (Nominal *P*_75% cutoff_ = 3.30 × 10^−3^; FDR_75% cutoff_ = 4.65 × 10^−2^). Biological pathways with an FDR less than 0.1 included nuclear transcription factor peroxisome proliferator-activated receptor (PPAR) signaling (Nominal *P*_75% cutoff_ = 3.50 × 10^−3^; FDR_75% cutoff_ = 6.63 × 10^−2^) and interleukin-6 (IL-6) signaling (Nominal *P*_75% cutoff_ = 7.30 × 10^−3^; FDR_75% cutoff_ = 8.61 × 10^−2^), both of which may be associated with MDD^22–24^.

**Table 2:** Lists of SNPs, and their closest genes, showing genome-wide significant association, or suggestive significant association, with psychological distress (assessed by the K6 scale; cut-off point: 5)

SNP	CHR	BP	Closest gene	A1	A2	FRQ	OR	95% CI	*P*	genotyped or imputed
rs6073833	20	44279894	WFDC11	T	G	0.6832	1.2027	1.1402–1.2652	7.60E-09	imputed
rs11752111	6	77877447	HTR1B	T	C	0.6938	0.8646	0.8023–0.9269	4.92E-06	imputed
rs58341733	3	193460070	OPA1	G	A	0.9031	1.2693	1.1662–1.3724	5.86E-06	imputed
rs78163065	1	12907449	NSM	C	G	0.8801	1.3949	1.2508–1.5390	5.90E-06	imputed
rs117241091	6	95246454	TSG1	G	A	0.9754	0.6103	0.3961–0.8245	6.24E-06	imputed
rs74930492	6	156076807	NOX3	C	T	0.5123	0.8748	0.8168–0.9328	6.39E-06	imputed

SNPs with genome wide significance (*P* < 5.00 × 10^−8^) were highlighted in pink. List includes SNPs located within linkage disequilibrium blocks

SNP variant identifier, *CHR* chromosome code, *BP* base-pair coordinate, *A1* allele 1 (effect allele), *A2* allele 2, *FRQ* allele 1 frequency, *OR* odds ratio, *95% CI* 95% confidence interval, *P* association test *p* value

Next, we evaluated whether the loci observed in the present GeneQuest (GQ) psychological distress GWAS were replicated in the results based on the previous GWASs. In particular, we assessed the extent to which loci with a P value less than 5.00 × 10^−5^ from the present GWAS were associated with phenotypes in the previous studies including PGC2 MDD (excluding 23andMe data)^6^, and GPC2 neuroticism GWAS^25–27^ (Supplementary Table 3). In this replication analysis, we faild to find association loci with a P value less than 0.05 in the PGC2 MDD GWAS. On the other hand, there were four loci that showed *P* values less than 0.05 in the GPC2 neuroticism study, with the same direction of effect. This may suggest risk loci shared between the psychological distress and neuroticism.

**Fig. 3:**
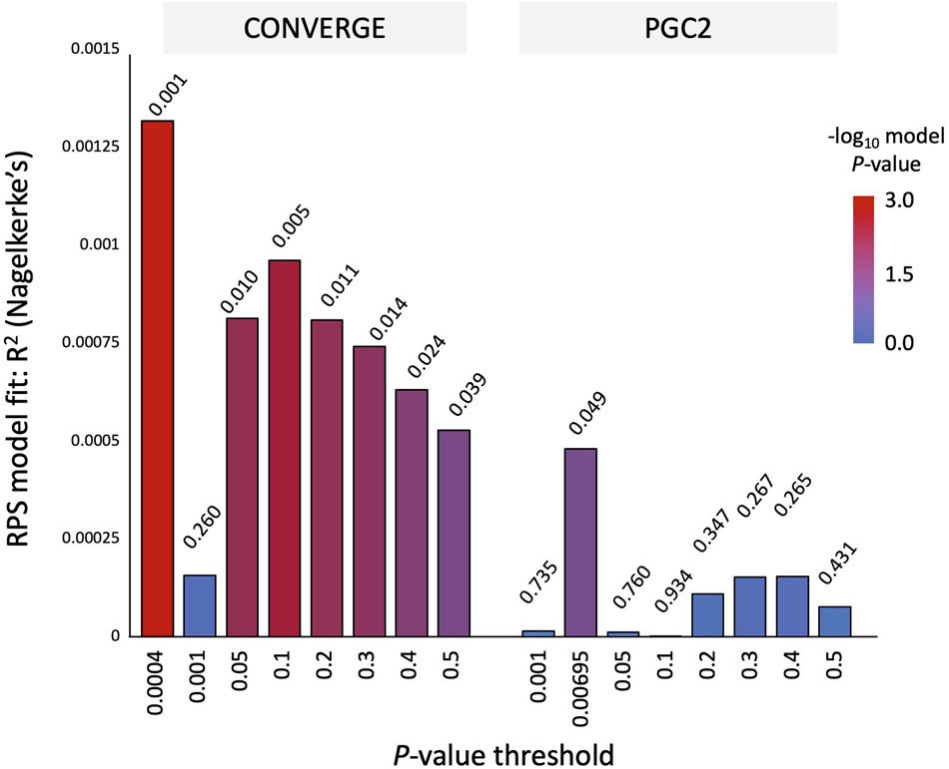
**Risk profile score** **(RPS) analysis.** The y-axis presents the Nagelkerke pseudo R^2^ that indicates the variance explained in psychological distress for each *P* value threshold shown. GeneQuest psychological distress (pruned data) was used as the target and the CONVERGE MDD or PGC2 MDD was used as the discovery

Finally, we evaluated shared genetic components between the present psychological distress GWAS results and those of the CONVERGE and PGC2 MDD GWASs based on risk profile score (RPS) analysis and genetic correlation analysis. In the RPS analysis, we set discovery (training) datasets obtained from PGC2 and CONVERGE, whereas training dataset from the current GQ GWAS, in which 229,276 SNPs, were obtained before imputation (Fig. 3). We found a significant replication in the RPS scores of the GQ case/control status based on the CONVERGE (the highest *R*^2^ = 0.0013, best *P* *=* 0.001, *P*-value threthold = 0.0004) dataset, but no replication based on the PGC2 MDD (the highest *R*^2^ = 0.00044, best *P* *=* 0.049, *P*-value threthold = 0.00695) was observed. We also assessed genetic correlations between the GQ psychological distress results and other GWAS results including CONVERGE MDD, PGC2 MDD, and GPC2 neuroticism results (Table 3). For CONVERGE results, similar ancestry (East Asian) was evaluated, thus LD score regression (LDSC) analysis^19^ was suitable. However, for the GWASs based on European populations (PGC2 MDD and GPC2 neuroticism GWASs), it is difficult to calculate the genetic correlation between different populations with LDSC analysis. Therefore, we used recently developed software, Popcorn, to analyze trans-population genetic correlation. Based on the LDSC analysis for CONVERGE, we observed a non-significant trend (*P* = 0.0529) for the estimate of the genetic correlation (*r*_*g*_: 0.368). However, based on the Popcorn analysis, significant correlations were observed between our Japanese samples and the PGC2 MDD GWASs (*P* for |*ρ*_ge_| > 0: 4.15 × 10^−4^, *P* for |*ρ*_gi_| > 0: 3.28 × 10^−7^, *P* for |*ρ*_ge_| < 1: 2.68 × 10^−7^, *P* for |*ρ*_gi_| < 1: 9.27 × 10^−10^; *ρ*_ge_ = 0.394, *ρ*_gi = _0.358), though there were no significant correlations with the GPC2 neuroticism GWAS (*P* for |*ρ*_ge_| > 0: 0.0787, *P* for |*ρ*_gi_| > 0: 0.0817, *P* for |*ρ*_ge_| < 1: 0.179, *P* for |*ρ*_gi_| < 1: 0.243; *ρ*_ge = _0.512, *ρ*_gi_ = 0.544).

**Table 3:** Genetic correlation assay for GeneQuest psychological distress GWAS

sample compared	MDD (CONVERGE)	MDD (PGC2)	Neuroticism (GPC2)
ethnicity	EAS	EUR	EUR
r_g_	0.368		
SE	0.190		
*P*	0.0529		
ρ_ge_		0.394	0.512
SE		0.118	0.362
*P* for |ρ_ge_| > 0*		**4.15E-04**	0.0787
*P* for |ρ_ge_| < 1**		**2.68E-07**	0.179
ρ_gi_		0.358	0.544
SE		0.105	0.391
*P* for |ρ_gi_| > 0*		**3.28E-04**	0.0817
*P* for |ρ_gi_| < 1**		**9.27E-10**	0.243

*EAS* East Asian ancestries, *EUR* European ancestries, *MDD* major depressive disorder, *r*_*g*_ estimated genetic correlation, *SE* standard error, *ρ*_*ge*_ genetic effect correlation (the correlation coefficient of the population-specific allele-variance-normalized SNP effect sizes), *ρ*_*gi*_ genetic impact correlation (the correlation coefficient of the per-allele SNP effect sizes)

*test that the genetic correlation is greater than 0 (one-sided)

**test that the genetic correlation is less than 1.0

## Discussion

In the present GWAS, loci associated with psychological distress were assessed in a Japanese population and a genome-wide significant association with psychological distress was observed at 20q13.12. The linkage disequilibrium block (LDB) to which the top-hit SNP belongs contains a few genes, including *WFDC11, 10B*, and *9*, and we cannot identify the causal gene(s) for psychological distress. However, there are studies reported that association of *WFDC11* with psychiatric disorder. In particular, *WFDC11* expression was up-regulated in autism^28^ and *WFDC11* methylation levels were down-regulated in schizophrenia^29^. WFDCs were first identified as protease inhibitors, but the WFDC11 target molecules have not been identified. While their precise molecular functions have not been fully elucidated, recent studies demonstrated that proteins with a WFDC domain may be involved in inflammation-related signaling^30–32^. For example, *WFDC12* is known to induce down-regulation of the pro-inflammatory signal in monocytes^32^. This is consistent with the hypothesis that immune dysregulation-related states, such as chronic inflammation and immunosuppression, might be involved in the pathogenesis of MDD^33–35^. It is, however, unclear whether *WFDC11*, *10B*, and *9* are involved in the pathophysiology of psychological distress via its regulatory roles in inflammation and/or immune state^36^. Assessing the molecular functions of the WFDCs, in inflammation models of depression in rodents may help reveal whether WFDC11 plays a role in the pathophysiology of psychological distress/MDD. The top-ranked gene-sets detected by gene set enrichment analysis included those involved in IL-6 and PPAR signaling, both of which have been associated with MDD/psychological distress. In particular, IL-6 is one of the most consistent inflammatory markers elevated in MDD^23,24^. PPAR-γ agonists were reported to have antidepressant properties, and PPAR is thought to represent a potential molecular target for MDD treatment^22^. This supports the external validity of the study. Significant enrichments were also detected for the PDGF and 14-3-3 signaling. While there are currently no reports indicating their significant associations with psychological distress/MDD, those signaling pathways may be involved in the pathophysiology of psychological distress/MDD.

To replicate our top-hit and confirm the shared genetic components with MDD or neuroticism, we consulted GWAS results based on other datasets from similar and different populations. We assessed whether there are replications of risk loci found in the present and previous studies. No nearby locus for our top-hit showed an association (*P* < 0.05) with MDD in the PGC2 study (Supplementary Table 4) or the CONVERGE study (data not shown), which was performed on an East Asian population (Han Chinese population). Thus, no replication was obtained for the top-hit. These discrepancies may be due to the differences in genetic background among studies and/or in environmental exposures. On the other hand, several top-hit loci in SNPs from the WFDC cluster display weak associations with neuroticism (Supplementary Table 5), suggesting that risk locus may be shared between psychological distress and neuroticism.

In association analysis, where we assessed the extent to which psychological distress case-control status in the present GWAS can be predicted by MDD RPSs. The best *P* for the CONVERGE MDD GWAS was barely significant, while that for the PGC2 MDD GWAS failed to reach the significant level. This may be due to the influences of LD differences and/or unique risks for GQ subjects. For the genetic correlation analysis, that non-significant trend for the genetic correlation with CONVERGE was observed, while PGC2 MDD GWAS from EUR showed a significant correlation. This might be derived from the sample size differences as PGC2 analyzed the largest sample size, but CONVERGE targeted less than 1/5~1/10^7,25–27^. However, we assume that our psychological distress GWAS shared the genetic component for MDD with the EAS or EUR populations. A larger sample size is essential for conclusive results, but our sample captured an appropriate phenotype related to MDD.

There are a some limitations, that are noted, to our present study. First, given the expected small effect sizes of the associated variants for MDD or psychological distress^6^, the sample size of approximately ten thousand may not be sufficient for precisely assessing risk variants. Indeed, top-hit locus reported in the PGC2 MDD study did not show significant overlap with the loci identified in the present GWAS, and the effect sizes of the detected top-hit loci in the preset study were larger in either direction when compared to those reported in the PGC2 MDD study. In addition, the effect of environmental factors was not assessed in the present study, although gene-environment interactions are thought to play a major role in the susceptibility and pathogenesis of depressive state/MDD. These factors may reduce the power of the study. Second, there can be a selection bias in participant recruitment, as the subjects voluntarily participated in the project, a preference for novelty may be higher in the participants than in the general population. Thus, there can be biased genotype distribution in the subjects, which may also affect the power of the GWAS. Additionally, the trait selection was carried out with self-rating questionnaires, not with psychiatric consultation. In summary, we identified a locus as a novel susceptibility region associated with psychological distress in the Japanese population. Further replication is necessary to confirm the present findings and to uncover the genetic landscape for psychological distress traits.

## Supplementary information

Supplemental Table 1

Supplemental Table 2

Supplemental Table 3

Supplemental Table 4

Supplemental Table 5

## References

[1] Ebmeier KP, Donaghey C, Steele JD (2006). Recent developments and current controversies in depression. Lancet Lond. Engl..

[2] Kupfer DJ, Frank E, Phillips ML (2012). Major depressive disorder: new clinical, neurobiological, and treatment perspectives. Lancet.

[3] Cross-Disorder Group of the Psychiatric Genomics Consortium. (2013). Identification of risk loci with shared effects on five major psychiatric disorders: a genome-wide analysis. Lancet Lond. Engl..

[4] Hyde CL (2016). Identification of 15 genetic loci associated with risk of major depression in individuals of European descent. Nat. Genet..

[5] Direk N (2017). An analysis of two genome-wide association meta-analyses identifies a new locus for broad depression phenotype. Biol. Psychiatry.

[6] Wray NR (2018). Genome-wide association analyses identify 44 risk variants and refine the genetic architecture of major depression. Nat. Genet..

[7] Converge Consortium. (2015). Sparse whole-genome sequencing identifies two loci for major depressive disorder. Nature.

[8] Ikeda M (2016). Genome-wide environment interaction between depressive state and stressful life events. J. Clin. Psychiatry.

[9] Gillan CM, Daw ND (2016). Taking psychiatry research online. Neuron.

[10] Asano, et al. Annual conference of informatics in biology, medicine and pharmacology. (2015).

[11] Okamoto, et al. Annual conference of informatics in biology, medicine and pharmacology. (2015).

[12] Sakurai K, Nishi A, Kondo K, Yanagida K, Kawakami N (2011). Screening performance of K6/K10 and other screening instruments for mood and anxiety disorders in Japan. Psychiatry Clin. Neurosci..

[13] Delaneau O, Marchini J, Zagury JF (2012). A linear complexity phasing method for thousands of genomes. Nat. Methods.

[14] Howie, B. N., Donnelly, P. & Marchini, J. A flexible and accurate genotype imputation method for the next generation of genome-wide association studies. PLoS Genet. 5, (2009).. 10.1371/journal.pgen.1000529 PMC2689936 19543373

[15] 1000 Genomes Project Consortium. (2015). A global reference for human genetic variation. Nature.

[16] Segrè, A. V. et al. Common inherited variation in mitochondrial genes is not enriched for associations with type 2 diabetes or related glycemic traits. *PLoS Genet*. **6**, e1001058 (2010). 10.1371/journal.pgen.1001058 PMC2920848 20714348

[17] Ikeda M (2018). A genome-wide association study identifies two novel susceptibility loci and trans population polygenicity associated with bipolar disorder. Mol. Psychiatry.

[18] Euesden J, Lewis CM, O’Reilly PF (2015). PRSice: Polygenic Risk Score software. Bioinformatics.

[19] Zheng J (2017). LD Hub: a centralized database and web interface to perform LD score regression that maximizes the potential of summary level GWAS data for SNP heritability and genetic correlation analysis. Bioinformatics.

[20] Brown BC (2016). Asian genetic epidemiology network type 2 diabetes consortium, Ye, C. J., Price, A. L. & Zaitlen, N. Transethnic genetic-correlation estimates from summary statistics. Am. J. Hum. Genet..

[21] Kessler RC (2002). The categorical versus dimensional assessment controversy in the sociology of mental illness. J. Health Soc. Behav..

[22] Colle R (2017). PPAR-γ agonists for the treatment of major depression: a review. Pharmacopsychiatry.

[23] Dowlati Y (2010). A meta-analysis of cytokines in major depression. Biol. Psychiatry.

[24] Yang K (2007). Levels of serum interleukin (IL)-6, IL-1beta, tumour necrosis factor-alpha and leptin and their correlation in depression. Aust. N. Z. J. Psychiatry.

[25] de Moor MHM (2012). Meta-analysis of genome-wide association studies for personality. Mol. Psychiatry.

[26] van den Berg SM (2014). Harmonization of neuroticism and extraversion phenotypes across inventories and cohorts in the genetics of personality consortium: an application of item response theory. Behav. Genet..

[27] Genetics of Personality Consortium. (2015). Meta-analysis of genome-wide association studies for neuroticism, and the polygenic association with major depressive disorder. JAMA Psychiatry.

[28] Griesi-Oliveira K (2015). Modeling non-syndromic autism and the impact of TRPC6 disruption in human neurons. Mol. Psychiatry.

[29] Horvath S (2012). Aging effects on DNA methylation modules in human brain and blood tissue. Genome Biol..

[30] Scott A, Weldon S, Taggart CC (2011). SLPI and elafin: multifunctional antiproteases of the WFDC family. Biochem. Soc. Trans..

[31] Ressler SJ (2014). WFDC1 is a key modulator of inflammatory and wound repair responses. Am. J. Pathol..

[32] Glasgow AMA (2015). A role for whey acidic protein four-disulfide-core 12 (WFDC12) in the regulation of the inflammatory response in the lung. Thorax.

[33] Barnes J, Mondelli V, Pariante CM (2017). Genetic contributions of inflammation to depression. Neuropsychopharmacology.

[34] Miller AH, Raison CL (2016). The role of inflammation in depression: from evolutionary imperative to modern treatment target. Nat. Rev. Immunol..

[35] Dantzer R, O’Connor JC, Freund GG, Johnson RW, Kelley KW (2008). From inflammation to sickness and depression: when the immune system subjugates the brain. Nat. Rev. Neurosci..

[36] López-Figueroa AL (2004). Serotonin 5-HT1A, 5-HT1B, and 5-HT2A receptor mRNA expression in subjects with major depression, bipolar disorder, and schizophrenia. Biol. Psychiatry.

