# Supplementary material for: Genome-wide association study identifies a novel locus associated with psychological distress in the Japanese population
Source: Transl Psychiatry. 2019 Jan 31;9:52. doi: 10.1038/s41398-019-0383-z (PMC6355763; doi:10.1038/s41398-019-0383-z)
Supplement: Supplementary file 1 — Supplemental Table 1 [file 41398_2019_383_MOESM1_ESM.pdf]

Supplementary Table 1. Lists of SNPs showing genome-wide significant association, or suggestive significant association, with psychological distress and their closest genes.

| SNP | CHR | BP | A1 | A2 | FRQ | OR | SE | P |
|---|---|---|---|---|---|---|---|---|
| rs6073833 | 20 | 44279894 | T | G | 0.6832 | 1.2027 | 0.0319 | 7.60E-09 |
| rs6073859 | 20 | 44321884 | G | A | 0.6832 | 1.2022 | 0.0319 | 8.02E-09 |
| rs6073843 | 20 | 44293956 | G | A | 0.6791 | 1.2004 | 0.0317 | 8.04E-09 |
| rs6073842 | 20 | 44293841 | G | C | 0.6791 | 1.2004 | 0.0317 | 8.04E-09 |
| rs6104290 | 20 | 44321902 | G | T | 0.6832 | 1.202 | 0.0319 | 8.29E-09 |
| rs13041338 | 20 | 44283123 | G | A | 0.678 | 1.1992 | 0.0316 | 8.92E-09 |
| rs6065854 | 20 | 44288816 | T | C | 0.6777 | 1.1991 | 0.0316 | 8.94E-09 |
| rs1586439 | 20 | 44287425 | T | C | 0.6777 | 1.199 | 0.0316 | 9.03E-09 |
| rs6073835 | 20 | 44286454 | C | T | 0.6777 | 1.199 | 0.0316 | 9.04E-09 |
| rs1825777 | 20 | 44287194 | A | G | 0.6777 | 1.199 | 0.0316 | 9.05E-09 |
| rs6065852 | 20 | 44280955 | C | A | 0.6771 | 1.1997 | 0.0317 | 9.06E-09 |
| rs6104285 | 20 | 44295416 | T | C | 0.6777 | 1.1988 | 0.0316 | 9.22E-09 |
| rs11696685 | 20 | 44295134 | A | G | 0.6779 | 1.1989 | 0.0316 | 9.24E-09 |
| rs6104283 | 20 | 44291410 | A | G | 0.6789 | 1.1995 | 0.0317 | 9.25E-09 |
| rs6065853 | 20 | 44284023 | C | A | 0.6777 | 1.1988 | 0.0316 | 9.28E-09 |
| rs6065863 | 20 | 44316083 | G | T | 0.6773 | 1.1981 | 0.0315 | 1.00E-08 |
| rs6104273 | 20 | 44280726 | T | C | 0.6799 | 1.199 | 0.0317 | 1.02E-08 |
| rs6073834 | 20 | 44279989 | C | A | 0.6799 | 1.199 | 0.0317 | 1.02E-08 |
| rs6130877 | 20 | 44280385 | C | T | 0.68 | 1.1991 | 0.0317 | 1.02E-08 |
| rs6065851 | 20 | 44279034 | C | T | 0.68 | 1.199 | 0.0317 | 1.03E-08 |
| rs6073850 | 20 | 44301084 | C | T | 0.6772 | 1.1979 | 0.0315 | 1.04E-08 |
| rs6104276 | 20 | 44284591 | T | G | 0.6781 | 1.1983 | 0.0316 | 1.06E-08 |
| rs6073840 | 20 | 44289370 | A | G | 0.6837 | 1.2012 | 0.032 | 1.06E-08 |
| rs6073848 | 20 | 44298550 | G | A | 0.6769 | 1.1975 | 0.0315 | 1.06E-08 |
| rs6094192 | 20 | 44297796 | G | A | 0.6769 | 1.1975 | 0.0315 | 1.08E-08 |
| rs6065859 | 20 | 44302697 | C | T | 0.6769 | 1.1974 | 0.0315 | 1.08E-08 |
| rs6073849 | 20 | 44299385 | A | T | 0.6769 | 1.1974 | 0.0315 | 1.09E-08 |
| rs6073846 | 20 | 44296323 | A | C | 0.677 | 1.1975 | 0.0315 | 1.10E-08 |
| rs1487319 | 20 | 44295943 | A | G | 0.677 | 1.1975 | 0.0315 | 1.11E-08 |
| rs3746596 | 20 | 44312028 | C | T | 0.6772 | 1.1974 | 0.0315 | 1.11E-08 |
| rs6065846 | 20 | 44268086 | T | A | 0.6805 | 1.198 | 0.0316 | 1.14E-08 |
| rs1013562 | 20 | 44310744 | T | C | 0.6771 | 1.1971 | 0.0315 | 1.15E-08 |
| rs2072974 | 20 | 44313401 | G | A | 0.6771 | 1.1971 | 0.0315 | 1.15E-08 |
| rs2281211 | 20 | 44312858 | C | T | 0.6771 | 1.1971 | 0.0315 | 1.16E-08 |
| rs6073855 | 20 | 44309670 | C | T | 0.6769 | 1.197 | 0.0315 | 1.16E-08 |
| rs8123346 | 20 | 44309987 | T | A | 0.6769 | 1.197 | 0.0315 | 1.16E-08 |
| rs6073856 | 20 | 44313020 | C | T | 0.6769 | 1.197 | 0.0315 | 1.16E-08 |
| rs6065861 | 20 | 44311056 | A | G | 0.6769 | 1.1969 | 0.0315 | 1.16E-08 |
| rs1487310 | 20 | 44308789 | T | C | 0.6769 | 1.1969 | 0.0315 | 1.16E-08 |
| rs6073851 | 20 | 44306978 | C | T | 0.6769 | 1.1969 | 0.0315 | 1.16E-08 |
| rs6124726 | 20 | 44307866 | T | A | 0.6769 | 1.1969 | 0.0315 | 1.16E-08 |
| rs6073854 | 20 | 44309605 | A | G | 0.6769 | 1.1969 | 0.0315 | 1.16E-08 |
| rs4810465 | 20 | 44311354 | C | T | 0.6769 | 1.1969 | 0.0315 | 1.16E-08 |
| rs6073844 | 20 | 44294234 | G | A | 0.681 | 1.1986 | 0.0318 | 1.18E-08 |
| rs147282660 | 20 | 44317477 | A | G | 0.6874 | 1.202 | 0.0323 | 1.24E-08 |
| rs6073831 | 20 | 44279550 | A | T | 0.6821 | 1.1988 | 0.0318 | 1.24E-08 |
| rs6073860 | 20 | 44321946 | C | A | 0.6863 | 1.2005 | 0.0321 | 1.27E-08 |
| rs6104298 | 20 | 44327774 | A | G | 0.6822 | 1.1989 | 0.0319 | 1.37E-08 |
| chr20:44299878:D | 20 | 44299878 | AG | A | 0.6783 | 1.1965 | 0.0316 | 1.38E-08 |
| rs4812955 | 20 | 44324638 | G | C | 0.6798 | 1.1969 | 0.0317 | 1.43E-08 |
| rs6073820 | 20 | 44249272 | T | A | 0.6795 | 1.196 | 0.0316 | 1.44E-08 |
| chr20:44322603:D | 20 | 44322603 | CA | C | 0.6813 | 1.1976 | 0.0318 | 1.44E-08 |
| chr20:44270633:D | 20 | 44270633 | TC | T | 0.6792 | 1.1958 | 0.0316 | 1.46E-08 |
| rs2171371 | 20 | 44273482 | T | C | 0.6792 | 1.1958 | 0.0316 | 1.46E-08 |
| rs7266372 | 20 | 44266635 | T | C | 0.6792 | 1.1957 | 0.0316 | 1.47E-08 |
| rs6094184 | 20 | 44256575 | G | A | 0.6792 | 1.1957 | 0.0316 | 1.47E-08 |
| rs6073827 | 20 | 44265556 | T | C | 0.6792 | 1.1957 | 0.0316 | 1.47E-08 |
| rs6104252 | 20 | 44254323 | G | A | 0.6792 | 1.1957 | 0.0316 | 1.47E-08 |
| rs6104294 | 20 | 44326850 | T | C | 0.6798 | 1.1968 | 0.0317 | 1.47E-08 |
| rs6104249 | 20 | 44250487 | C | G | 0.6792 | 1.1957 | 0.0316 | 1.47E-08 |
| rs6104250 | 20 | 44251479 | C | T | 0.6792 | 1.1957 | 0.0316 | 1.47E-08 |
| rs4812938 | 20 | 44249919 | T | A | 0.6792 | 1.1957 | 0.0316 | 1.47E-08 |
| rs6073821 | 20 | 44251791 | A | G | 0.6792 | 1.1957 | 0.0316 | 1.47E-08 |
| rs6104295 | 20 | 44326917 | C | T | 0.6798 | 1.1968 | 0.0317 | 1.48E-08 |
| rs6104293 | 20 | 44326298 | T | C | 0.6798 | 1.1967 | 0.0317 | 1.48E-08 |

| | | | | | | | | |
|---|---|---|---|---|---|---|---|---|
| **rs1487325** | **20** | **44258989** | **G** | **A** | **0.6794** | **1.1958** | **0.0316** | **1.48E-08** |
| **rs6073861** | **20** | **44325708** | **G** | **A** | **0.6798** | **1.1967** | **0.0317** | **1.48E-08** |
| **rs6104291** | **20** | **44323368** | **C** | **G** | **0.6797** | **1.1966** | **0.0317** | **1.48E-08** |
| **rs6065866** | **20** | **44323440** | **G** | **A** | **0.6797** | **1.1966** | **0.0317** | **1.48E-08** |
| **rs6130893** | **20** | **44322582** | **A** | **C** | **0.6797** | **1.1966** | **0.0317** | **1.49E-08** |
| **rs6130894** | **20** | **44322673** | **G** | **A** | **0.6797** | **1.1966** | **0.0317** | **1.49E-08** |
| **rs6124727** | **20** | **44325623** | **T** | **C** | **0.6784** | **1.196** | **0.0317** | **1.66E-08** |
| **rs6104247** | **20** | **44244938** | **C** | **T** | **0.6795** | **1.195** | **0.0316** | **1.70E-08** |
| **rs2235598** | **20** | **44238122** | **G** | **A** | **0.6821** | **1.1966** | **0.0318** | **1.71E-08** |
| **rs2235600** | **20** | **44238299** | **T** | **G** | **0.6795** | **1.195** | **0.0316** | **1.71E-08** |
| **rs978778** | **20** | **44243376** | **C** | **A** | **0.6796** | **1.1951** | **0.0316** | **1.71E-08** |
| **rs2281210** | **20** | **44243172** | **T** | **G** | **0.6793** | **1.195** | **0.0316** | **1.72E-08** |
| **rs6073825** | **20** | **44258217** | **T** | **C** | **0.6791** | **1.1947** | **0.0316** | **1.73E-08** |
| **rs6073815** | **20** | **44234615** | **A** | **T** | **0.6802** | **1.1952** | **0.0316** | **1.75E-08** |
| **rs6130896** | **20** | **44327532** | **A** | **G** | **0.6873** | **1.1992** | **0.0322** | **1.76E-08** |
| **rs4812933** | **20** | **44230792** | **C** | **A** | **0.6798** | **1.1953** | **0.0317** | **1.81E-08** |
| **rs13041885** | **20** | **44283477** | **G** | **T** | **0.6829** | **1.1964** | **0.0319** | **1.85E-08** |
| **rs6094197** | **20** | **44316492** | **C** | **T** | **0.6817** | **1.1973** | **0.0321** | **1.98E-08** |
| **rs2272961** | **20** | **44259549** | **G** | **A** | **0.6784** | **1.1934** | **0.0315** | **2.06E-08** |
| **rs4812953** | **20** | **44322248** | **G** | **T** | **0.6822** | **1.1954** | **0.0319** | **2.12E-08** |
| **rs6073788** | **20** | **44213773** | **C** | **T** | **0.6799** | **1.1939** | **0.0317** | **2.24E-08** |
| **rs2206449** | **20** | **44230292** | **C** | **A** | **0.3206** | **0.8379** | **0.0317** | **2.31E-08** |
| **rs6073784** | **20** | **44201931** | **T** | **A** | **0.68** | **1.193** | **0.0317** | **2.55E-08** |
| **rs6130865** | **20** | **44211845** | **C** | **T** | **0.6801** | **1.1926** | **0.0317** | **2.74E-08** |
| **rs2093090** | **20** | **44205464** | **C** | **T** | **0.6801** | **1.1923** | **0.0317** | **2.87E-08** |
| **rs6094177** | **20** | **44231419** | **A** | **T** | **0.6794** | **1.1942** | **0.0322** | **3.58E-08** |
| **chr20:44231415:D** | **20** | **44231415** | **TGTGA** | **T** | **0.7687** | **1.2418** | **0.0393** | **3.65E-08** |
| **rs4812948** | **20** | **44282409** | **G** | **C** | **0.6892** | **1.1928** | **0.032** | **3.68E-08** |
| **rs6104297** | **20** | **44327621** | **A** | **G** | **0.7044** | **1.2015** | **0.0334** | **3.97E-08** |
| **rs6104257** | **20** | **44262100** | **G** | **T** | **0.6996** | **1.1962** | **0.0327** | **4.17E-08** |
| **rs6073838** | **20** | **44289156** | **C** | **A** | **0.7214** | **1.2079** | **0.0345** | **4.23E-08** |
| **rs4812909** | **20** | **44167244** | **G** | **A** | **0.692** | **1.1932** | **0.0322** | **4.27E-08** |
| **rs6073839** | **20** | **44289328** | **G** | **A** | **0.7127** | **1.2061** | **0.0342** | **4.27E-08** |
| **rs4810466** | **20** | **44322266** | **C** | **T** | **0.7036** | **1.2008** | **0.0335** | **4.83E-08** |
| rs6073822 | 20 | 44251880 | A | G | 0.6754 | 1.1867 | 0.0315 | 5.52E-08 |
| chr20:44231417:D | 20 | 44231417 | TGA | T | 0.7574 | 1.2288 | 0.0379 | 5.63E-08 |
| rs6073823 | 20 | 44256910 | G | C | 0.5319 | 1.1847 | 0.0313 | 6.24E-08 |
| chr20:44296110:D | 20 | 44296110 | TA | T | 0.6914 | 1.189 | 0.0322 | 7.68E-08 |
| rs6124724 | 20 | 44281525 | A | G | 0.6331 | 1.1922 | 0.0328 | 8.11E-08 |
| rs6073841 | 20 | 44290421 | C | T | 0.7019 | 1.2049 | 0.035 | 1.01E-07 |
| rs6065865 | 20 | 44322016 | G | T | 0.701 | 1.195 | 0.0336 | 1.10E-07 |
| rs6104278 | 20 | 44285991 | G | A | 0.7261 | 1.2045 | 0.0351 | 1.13E-07 |
| chr20:44222856:D | 20 | 44222856 | AGACCTGGTGGT | A | 0.7021 | 1.192 | 0.0331 | 1.15E-07 |
| rs6130870 | 20 | 44263263 | T | C | 0.7059 | 1.1919 | 0.0333 | 1.37E-07 |
| rs6124722 | 20 | 44263264 | G | A | 0.7059 | 1.1919 | 0.0333 | 1.37E-07 |
| chr20:44252266:I | 20 | 44252266 | C | CA | 0.6988 | 1.1877 | 0.0329 | 1.73E-07 |
| rs146537075 | 20 | 44321432 | G | A | 0.6999 | 1.1905 | 0.0334 | 1.74E-07 |
| rs714595 | 20 | 44330591 | G | A | 0.7174 | 1.1899 | 0.0336 | 2.24E-07 |
| rs6073862 | 20 | 44331705 | T | C | 0.724 | 1.1959 | 0.0346 | 2.25E-07 |
| rs6130897 | 20 | 44330004 | G | A | 0.7173 | 1.1896 | 0.0336 | 2.31E-07 |
| rs6094199 | 20 | 44321057 | A | T | 0.7172 | 1.1907 | 0.0344 | 3.86E-07 |
| rs6073774 | 20 | 44150077 | C | A | 0.6724 | 1.1734 | 0.0316 | 4.21E-07 |
| rs6130855 | 20 | 44148515 | T | C | 0.6699 | 1.172 | 0.0316 | 5.16E-07 |
| rs6130878 | 20 | 44281442 | A | G | 0.7309 | 1.1936 | 0.0357 | 7.32E-07 |
| chr20:44362545:I | 20 | 44362545 | T | TGTGTATA | 0.8002 | 1.2142 | 0.0395 | 9.05E-07 |
| chr20:44362550:I | 20 | 44362550 | A | ATAGTG | 0.8058 | 1.2192 | 0.0405 | 9.73E-07 |
| rs6065830 | 20 | 44141024 | T | G | 0.6836 | 1.167 | 0.0317 | 1.14E-06 |
| chr20:44362548:I | 20 | 44362548 | G | GTATAGTGTA | 0.7902 | 1.2094 | 0.0393 | 1.32E-06 |
| rs6065827 | 20 | 44122560 | C | T | 0.6837 | 1.1652 | 0.0318 | 1.52E-06 |
| rs4812907 | 20 | 44140692 | T | C | 0.6849 | 1.165 | 0.0318 | 1.54E-06 |
| rs6073759 | 20 | 44115498 | A | T | 0.6814 | 1.1627 | 0.0319 | 2.23E-06 |
| rs11696176 | 20 | 44115425 | G | C | 0.6815 | 1.1626 | 0.0319 | 2.24E-06 |
| rs6130846 | 20 | 44115129 | A | G | 0.6815 | 1.1626 | 0.0319 | 2.28E-06 |
| rs6065823 | 20 | 44114873 | T | C | 0.6812 | 1.1623 | 0.0319 | 2.40E-06 |
| rs1883520 | 20 | 44130777 | T | C | 0.6855 | 1.1607 | 0.0317 | 2.68E-06 |
| rs6065829 | 20 | 44126608 | C | G | 0.6824 | 1.1599 | 0.0316 | 2.73E-06 |
| chr20:44113981:I | 20 | 44113981 | T | TA | 0.6825 | 1.1629 | 0.0322 | 2.76E-06 |
| rs2425747 | 20 | 44372893 | G | A | 0.7786 | 1.1858 | 0.0364 | 2.82E-06 |

| | | | | | | | | |
|---|---|---|---|---|---|---|---|---|
| rs2425746 | 20 | 44372620 | T | C | 0.7759 | 1.1835 | 0.036 | 2.83E-06 |
| rs74532911 | 1 | 71492133 | G | A | 0.9734 | 1.6587 | 0.1082 | 2.88E-06 |
| rs6073768 | 20 | 44130146 | A | G | 0.6855 | 1.1598 | 0.0317 | 2.99E-06 |
| rs6104282 | 20 | 44291055 | G | C | 0.732 | 1.1856 | 0.0365 | 3.15E-06 |
| rs9398156 | 6 | 108333900 | G | C | 0.9707 | 0.5993 | 0.1104 | 3.51E-06 |
| rs7634143 | 3 | 193478956 | C | T | 0.903 | 1.2755 | 0.0528 | 4.02E-06 |
| rs72779762 | 2 | 6943544 | A | T | 0.983 | 0.4037 | 0.1972 | 4.22E-06 |
| rs10145269 | 14 | 51769016 | C | T | 0.7493 | 0.8434 | 0.0372 | 4.66E-06 |
| rs6065856 | 20 | 44291022 | G | A | 0.7449 | 1.1795 | 0.0361 | 4.82E-06 |
| rs6032493 | 20 | 44364750 | G | A | 0.7575 | 1.1727 | 0.0348 | 4.83E-06 |
| rs11752111 | 6 | 77877447 | T | C | 0.6938 | 0.8646 | 0.0318 | 4.92E-06 |
| rs4551142 | 6 | 77865860 | T | A | 0.694 | 0.8648 | 0.0319 | 5.14E-06 |
| rs10943424 | 6 | 77860339 | C | T | 0.694 | 0.8649 | 0.0319 | 5.32E-06 |
| rs28896892 | 6 | 77867470 | T | C | 0.6938 | 0.8652 | 0.0319 | 5.50E-06 |
| rs58341733 | 3 | 193460070 | G | A | 0.9031 | 1.2693 | 0.0526 | 5.86E-06 |
| rs78163065 | 1 | 12907449 | C | G | 0.8801 | 1.3949 | 0.0735 | 5.90E-06 |
| rs117241091 | 6 | 95246454 | G | A | 0.9754 | 0.6103 | 0.1093 | 6.24E-06 |
| rs74930492 | 6 | 156076807 | C | T | 0.5123 | 0.8748 | 0.0296 | 6.39E-06 |
| rs6919606 | 6 | 77881855 | T | A | 0.6906 | 0.8666 | 0.0318 | 6.59E-06 |
| rs9384356 | 6 | 156073840 | C | T | 0.5133 | 0.8752 | 0.0296 | 6.74E-06 |
| rs232262 | 20 | 44350931 | C | G | 0.2465 | 0.8554 | 0.0347 | 6.77E-06 |
| rs9848957 | 3 | 193466666 | A | G | 0.9051 | 1.2682 | 0.0528 | 6.89E-06 |
| rs151169667 | 18 | 2325508 | A | C | 0.9877 | 0.4116 | 0.1975 | 6.96E-06 |
| rs6032468 | 20 | 44349105 | C | T | 0.2425 | 0.855 | 0.0349 | 7.05E-06 |
| rs232291 | 20 | 44334394 | A | C | 0.2244 | 0.8497 | 0.0363 | 7.09E-06 |
| rs232285 | 20 | 44338769 | T | C | 0.2242 | 0.8508 | 0.036 | 7.15E-06 |
| rs35997550 | 12 | 6297261 | C | T | 0.9691 | 0.5999 | 0.1139 | 7.22E-06 |
| rs232276 | 20 | 44343536 | G | A | 0.2245 | 0.8512 | 0.036 | 7.38E-06 |
| rs4708320 | 6 | 77874421 | T | C | 0.6927 | 0.867 | 0.0318 | 7.41E-06 |
| rs980984 | 20 | 44333052 | A | G | 0.246 | 0.8524 | 0.0357 | 7.45E-06 |
| rs232290 | 20 | 44334588 | C | G | 0.2246 | 0.8503 | 0.0362 | 7.58E-06 |
| rs36022418 | 6 | 77867927 | C | T | 0.7022 | 0.8645 | 0.0325 | 7.63E-06 |
| rs117793402 | 6 | 95145839 | T | C | 0.9815 | 0.5587 | 0.1301 | 7.65E-06 |
| rs10246388 | 7 | 39217813 | G | A | 0.0223 | 0.5059 | 0.1523 | 7.68E-06 |
| rs79216918 | 1 | 71431715 | T | C | 0.9714 | 1.5923 | 0.104 | 7.70E-06 |
| rs77440633 | 1 | 71417907 | T | C | 0.9714 | 1.5927 | 0.104 | 7.70E-06 |
| rs232270 | 20 | 44346426 | A | G | 0.2253 | 0.8513 | 0.036 | 7.76E-06 |
| rs232268 | 20 | 44347388 | T | C | 0.2256 | 0.8514 | 0.036 | 7.88E-06 |
| rs232267 | 20 | 44348034 | T | C | 0.2258 | 0.8516 | 0.036 | 8.04E-06 |
| rs10151462 | 14 | 51767550 | T | A | 0.7354 | 0.8494 | 0.0366 | 8.23E-06 |
| rs10139442 | 14 | 51767468 | G | C | 0.7354 | 0.8495 | 0.0366 | 8.31E-06 |
| rs11928384 | 3 | 193480560 | T | C | 0.9036 | 1.264 | 0.0526 | 8.33E-06 |
| rs6968349 | 7 | 31111617 | G | T | 0.3156 | 1.2277 | 0.0461 | 8.46E-06 |
| rs140887899 | 12 | 105694872 | G | A | 0.9897 | 3.3507 | 0.2716 | 8.53E-06 |
| rs2300163 | 1 | 71450277 | C | T | 0.9663 | 1.555 | 0.0993 | 8.68E-06 |
| rs117969002 | 6 | 95119959 | T | A | 0.9816 | 0.5593 | 0.1307 | 8.79E-06 |
| chr3:193460620:D | 3 | 193460620 | CA | C | 0.9039 | 1.2641 | 0.0527 | 8.83E-06 |
| rs131569 | 22 | 16880437 | G | A | 0.7264 | 0.7919 | 0.0525 | 8.86E-06 |
| chr6:77902086:D | 6 | 77902086 | ACT | A | 0.6911 | 0.8664 | 0.0323 | 8.89E-06 |
| rs77469813 | 1 | 247271108 | C | T | 0.9784 | 0.5349 | 0.1409 | 8.95E-06 |
| rs28572322 | 3 | 193463012 | G | C | 0.9036 | 1.2618 | 0.0524 | 8.96E-06 |
| rs7645962 | 3 | 193479055 | T | C | 0.9076 | 1.2682 | 0.0535 | 9.01E-06 |
| chr4:162704883:D | 4 | 162704883 | CT | C | 0.354 | 0.8639 | 0.033 | 9.12E-06 |
| rs147783933 | 18 | 22352252 | C | T | 0.9823 | 0.5716 | 0.1261 | 9.19E-06 |
| chr1:173370160:I | 1 | 173370160 | C | CAT | 0.5265 | 0.8679 | 0.0319 | 9.21E-06 |
| rs9322532 | 6 | 156026607 | G | A | 0.5604 | 0.8738 | 0.0304 | 9.33E-06 |
| rs2056161 | 20 | 44368763 | G | T | 0.7722 | 1.1698 | 0.0354 | 9.67E-06 |
| rs11924310 | 3 | 193480443 | C | G | 0.9038 | 1.2622 | 0.0526 | 9.68E-06 |
| rs7615525 | 3 | 193480424 | A | G | 0.9038 | 1.2621 | 0.0526 | 9.71E-06 |
| chr3:193481268:I | 3 | 193481268 | A | ACCTGCAGAGT | 0.9031 | 1.2626 | 0.0527 | 9.71E-06 |
| chr2:228699976:I | 2 | 228699976 | G | GT | 0.8386 | 1.2674 | 0.0536 | 9.78E-06 |
| rs232263 | 20 | 44350728 | A | G | 0.229 | 0.8535 | 0.0359 | 9.97E-06 |
| rs7637836 | 3 | 193480407 | G | A | 0.9038 | 1.2617 | 0.0526 | 1.00E-05 |

SNPs with genome wide significance (P < 5E-8) are shown in bold. List includes SNPs located within linkage disequilibrium blocks.
SNP: Variant identifier, CHR: Chromosome code, BP: Base-pair coordinate, A1: Allele 1 (effect allele), A2: Allele 2, FRQ: Allele 1 frequency, OR: odds ratio, SE: Standard error of effect estimate, P: Association test p-value
