# Supplementary material for: Genome-wide association study identifies a novel locus associated with psychological distress in the Japanese population
Source: Transl Psychiatry. 2019 Jan 31;9:52. doi: 10.1038/s41398-019-0383-z (PMC6355763; doi:10.1038/s41398-019-0383-z)
Supplement: Supplementary file 2 — Supplemental Table 2 [file 41398_2019_383_MOESM2_ESM.pdf]

Supplementary Table 2. Gene set enrichment analysis for GQ psychological distress GWAS.

Data base	Biological pathways/gene sets	75% Cut-off				95% Cut-off			
		NOMINAL GSEA P-value	FDR	Expected # of Genes	Observed # of Genes	NOMINAL GSEA P-value	FDR	Expected # of Genes	Observed # of Genes
Ingenuity	PDGF Signaling	5.00E-04	0.027	6	13	0.676	0.724	1	1
GOTERM	glucose metabolic process	1.30E-03	1.000	11	20	0.153	1.000	2	4
REACTOME	PACKAGING OF TELOMERE ENDS	1.50E-03	0.377	6	13	0.000	0.028	1	7
KEGG	VASCULAR SMOOTH MUSCLE CONTRACTION	2.80E-03	0.269	27	40	0.007	0.384	5	12
BIOCARTA	CARDIACEGF PATHWAY	2.80E-03	0.337	4	10	0.050	0.466	1	3
GOTERM	STAGA complex	3.00E-03	0.874	3	8	0.121	1.000	1	2
GOTERM	collagen fibril organization	3.30E-03	1.000	6	13	0.724	1.000	1	1
Ingenuity	14-3-3-mediated Signaling	3.30E-03	0.046	6	12	0.297	0.628	1	2
PANTHER_BIOLOGICAL_PROCESS	Porphyrin metabolism	3.50E-03	0.329	4	9	1.000	0.987	1	0
Ingenuity	PPAR Signaling	3.50E-03	0.066	4	10	0.583	0.682	1	1
KEGG	THYROID CANCER	4.10E-03	0.535	7	14	0.050	0.355	1	4
GOTERM	microtubule binding	4.20E-03	0.775	14	24	0.063	1.000	3	6
PANTHER_MOLECULAR_FUNCTION	Interferon	4.40E-03	0.232	2	5	0.033	0.444	0	2
GOTERM	muscle homeostasis	5.40E-03	1.000	3	8	0.483	1.000	1	1
GOTERM	kinetochore	5.50E-03	0.756	12	20	0.689	1.000	2	2
GOTERM	positive regulation of hormone secretion	6.70E-03	0.896	3	7	0.425	1.000	1	1
GOTERM	cytoskeleton	6.80E-03	0.750	158	183	0.691	1.000	32	29
GOTERM	cellular iron ion homeostasis	7.00E-03	0.795	7	13	0.391	1.000	1	2
Ingenuity	IL-6 Signaling	7.30E-03	0.086	7	13	0.386	0.602	1	2
GOTERM	microtubule cytoskeleton	7.80E-03	0.725	8	15	0.229	1.000	2	3
PANTHER_BIOLOGICAL_PROCESS	MAPKKK cascade	8.50E-03	0.719	39	53	0.048	0.614	8	13
GOTERM	dendritic spine	8.80E-03	0.765	9	16	0.112	1.000	2	4
KEGG	LEISHMANIA INFECTION	9.90E-03	0.320	13	21	0.262	0.662	3	4

Biological pathways/gene sets with an FDR of equal to or less than 0.05 are highlighted with pink, and those with an FDR of equal to or less than 0.10 are highlighted with blue. NOMINAL GESA P-value: nominal gene-set enrichment analysis P-values, FDR: false discovery rate; Data base: Gene Ontology term (GOTERM), Kyoto Encyclopedia of Genes and Genomes (KEGG), Protein Analysis Through Evolutionary Relationships (PANTHER), BioCarta, Reactome, and Ingenuity databases.
