# Supplementary material for: Genome-wide association study identifies a novel locus associated with psychological distress in the Japanese population
Source: Transl Psychiatry. 2019 Jan 31;9:52. doi: 10.1038/s41398-019-0383-z (PMC6355763; doi:10.1038/s41398-019-0383-z)
Supplement: Supplementary file 3 — Supplemental Table 3 [file 41398_2019_383_MOESM3_ESM.pdf]

Supplementary Table 3. List of locus showing genome-wide significant association (P < 5E-8), or suggestive significant association (P < 5E-6) in Chromosome 20 in the present GQ psychological distress GWAS and their OR, SE, and P value reported in previous GWASs.

			Psychological Distress (GQ)			MDD (PGC2 ex23andMe)			NEUROTICISM (GPC2)		
SNP	CHR	BP	OR	SE	P	OR	SE	P	BETA	SE	P
chr20:44113981:I	20	44113981	1.1629	0.0322	2.76E-06	1.00955	0.0109	3.84E-01			
rs6065823	20	44114873	1.1623	0.0319	2.40E-06	0.98817	0.0107	2.66E-01	0.0088	0.0079	2.67E-01
rs6130846	20	44115129	1.1626	0.0319	2.28E-06	0.98876	0.0107	2.93E-01	0.0089	0.0079	2.60E-01
rs11696176	20	44115425	1.1626	0.0319	2.24E-06	1.01106	0.0107	3.05E-01	-0.0091	0.0079	2.51E-01
rs6073759	20	44115498	1.1627	0.0319	2.23E-06	0.98896	0.0107	3.03E-01	0.009	0.0079	2.55E-01
rs6065827	20	44122560	1.1652	0.0318	1.52E-06	1.01147	0.0107	2.85E-01	-0.0096	0.0078	2.22E-01
rs6065829	20	44126608	1.1599	0.0316	2.73E-06	0.99064	0.0103	3.60E-01	0.0072	0.0074	3.32E-01
rs6073768	20	44130146	1.1598	0.0317	2.99E-06	0.98896	0.0107	2.97E-01	0.0071	0.0079	3.71E-01
rs1883520	20	44130777	1.1607	0.0317	2.68E-06	0.99114	0.0107	4.03E-01	0.0074	0.0077	3.38E-01
rs4812907	20	44140692	1.165	0.0318	1.54E-06	0.99173	0.0107	4.38E-01	0.0086	0.0077	2.65E-01
rs6065830	20	44141024	1.167	0.0317	1.14E-06	0.99045	0.0104	3.56E-01	0.0066	0.0074	3.72E-01
rs6130855	20	44148515	1.172	0.0316	5.16E-07	0.99144	0.0104	4.09E-01	0.0105	0.0076	1.66E-01
rs6073774	20	44150077	1.1734	0.0316	4.21E-07	1.00813	0.0107	4.47E-01	-0.0091	0.0077	2.36E-01
rs4812909	20	44167244	1.1932	0.0322	4.27E-08	1.00914	0.0108	3.98E-01	-0.007	0.0078	3.70E-01
rs6073784	20	44201931	1.193	0.0317	2.55E-08	1.01288	0.0106	2.26E-01	-0.0092	0.0077	2.31E-01
rs2093090	20	44205464	1.1923	0.0317	2.87E-08	1.01298	0.0107	2.29E-01	-0.0074	0.0078	3.46E-01
rs6130865	20	44211845	1.1926	0.0317	2.74E-08	1.01278	0.0107	2.37E-01	-0.0074	0.0078	3.44E-01
rs6073788	20	44213773	1.1939	0.0317	2.24E-08	1.01268	0.0105	2.32E-01	-0.0088	0.0077	2.51E-01
chr20:44222856:D	20	44222856	1.192	0.0331	1.15E-07	0.98807	0.0107	2.62E-01			
rs2206449	20	44230292	0.8379	0.0317	2.31E-08	0.9994	0.008	9.35E-01	0.0133	0.0057	2.08E-02
rs4812933	20	44230792	1.1953	0.0317	1.81E-08	1.01268	0.0105	2.32E-01	-0.0095	0.0077	2.17E-01
chr20:44231415:D	20	44231415	1.2418	0.0393	3.65E-08	0.97912	0.0139	1.29E-01	0.0072	0.008	3.68E-01
chr20:44231417:D	20	44231417	1.2288	0.0379	5.63E-08	1.01826	0.0133	1.74E-01			
rs6094177	20	44231419	1.1942	0.0322	3.58E-08	0.9856	0.0126	2.47E-01			
rs6073815	20	44234615	1.1952	0.0316	1.75E-08	0.98837	0.0105	2.67E-01	0.01	0.0077	1.94E-01
rs2235598	20	44238122	1.1966	0.0318	1.71E-08	1.01207	0.0105	2.54E-01	-0.0096	0.0077	2.13E-01
rs2235600	20	44238299	1.195	0.0316	1.71E-08	0.98807	0.0105	2.53E-01	0.0095	0.0077	2.16E-01
rs2281210	20	44243172	1.195	0.0316	1.72E-08	0.98797	0.0105	2.51E-01	0.0098	0.0077	2.03E-01
rs978778	20	44243376	1.1951	0.0316	1.71E-08	1.01278	0.0107	2.33E-01	-0.013	0.0086	1.30E-01
rs6104247	20	44244938	1.195	0.0316	1.70E-08	1.01227	0.0105	2.47E-01	-0.0099	0.0077	1.99E-01
rs6073820	20	44249272	1.196	0.0316	1.44E-08	1.01248	0.0105	2.37E-01	-0.0098	0.0077	2.05E-01
rs4812938	20	44249919	1.1957	0.0316	1.47E-08	1.01217	0.0105	2.51E-01	-0.0097	0.0077	2.10E-01
rs6104249	20	44250487	1.1957	0.0316	1.47E-08	0.98768	0.0105	2.40E-01	0.0098	0.0077	2.02E-01
rs6104250	20	44251479	1.1957	0.0316	1.47E-08	1.01197	0.0105	2.58E-01	-0.0093	0.0077	2.26E-01
rs6073821	20	44251791	1.1957	0.0316	1.47E-08	0.98817	0.0105	2.57E-01	0.0094	0.0077	2.23E-01
rs6073822	20	44251880	1.1867	0.0315	5.52E-08	0.98807	0.0105	2.53E-01	0.0096	0.0077	2.10E-01
chr20:44252266:I	20	44252266	1.1877	0.0329	1.73E-07	1.01308	0.0111	2.41E-01	-0.0096	0.0077	2.10E-01
rs6104252	20	44254323	1.1957	0.0316	1.47E-08	1.01207	0.0105	2.55E-01	-0.0097	0.0077	2.09E-01
rs6094184	20	44256575	1.1957	0.0316	1.47E-08	1.01207	0.0105	2.53E-01	-0.0093	0.0077	2.25E-01
rs6073823	20	44256910	1.1847	0.0313	6.24E-08	1.01949	0.0121	1.10E-01	0.0097	0.0077	2.10E-01
rs6073825	20	44258217	1.1947	0.0316	1.73E-08	0.98827	0.0105	2.63E-01	-0.0102	0.0077	1.87E-01
rs1487325	20	44258989	1.1958	0.0316	1.48E-08	1.01157	0.0105	2.76E-01	-0.0134	0.0084	1.09E-01
rs2272961	20	44259549	1.1934	0.0315	2.06E-08	1.01167	0.0105	2.71E-01	-0.0092	0.0077	2.36E-01
rs6104257	20	44262100	1.1962	0.0327	4.17E-08	1.01086	0.0105	3.06E-01	0.011	0.0081	1.77E-01
rs6130870	20	44263263	1.1919	0.0333	1.37E-07	0.98866	0.0108	2.93E-01	-0.011	0.0081	1.76E-01
rs6124722	20	44263264	1.1919	0.0333	1.37E-07	1.01147	0.0108	2.91E-01			
rs6073827	20	44265556	1.1957	0.0316	1.47E-08	0.98847	0.0105	2.70E-01	0.0097	0.0077	2.08E-01
rs7266372	20	44266635	1.1957	0.0316	1.47E-08	0.98837	0.0105	2.65E-01	0.0097	0.0077	2.07E-01
rs6065846	20	44268086	1.198	0.0316	1.14E-08	1.01147	0.0106	2.82E-01	-0.0095	0.0078	2.25E-01
chr20:44270633:D	20	44270633	1.1958	0.0316	1.46E-08	0.98926	0.0107	3.14E-01			
rs2171371	20	44273482	1.1958	0.0316	1.46E-08	0.98827	0.0105	2.61E-01	0.01	0.0077	1.92E-01
rs6065851	20	44279034	1.199	0.0317	1.03E-08	1.01167	0.0105	2.72E-01	-0.0117	0.0076	1.24E-01
rs6073831	20	44279550	1.1988	0.0318	1.24E-08	0.98807	0.0106	2.56E-01	0.0114	0.0077	1.39E-01
rs6073833	20	44279894	1.2027	0.0319	7.60E-09	0.98857	0.0105	2.76E-01	0.01	0.0076	1.93E-01
rs6073834	20	44279989	1.199	0.0317	1.02E-08	1.01157	0.0105	2.77E-01	-0.0099	0.0076	1.97E-01
rs6130877	20	44280385	1.1991	0.0317	1.02E-08	1.01147	0.0105	2.78E-01	-0.0102	0.0077	1.82E-01
rs6104273	20	44280726	1.199	0.0317	1.02E-08	0.98847	0.0105	2.71E-01	0.01	0.0076	1.90E-01
rs6065852	20	44280955	1.1997	0.0317	9.06E-09	1.01116	0.0097	2.53E-01	-0.011	0.0074	1.40E-01
rs6130878	20	44281442	1.1936	0.0357	7.32E-07	0.99035	0.0114	3.91E-01	0.0091	0.0085	2.81E-01

rs6124724	20	44281525	1.1922	0.0328	8.11E-08	0.99422	0.0107	5.86E-01	0.0086	0.0078	2.73E-01
rs4812948	20	44282409	1.1928	0.032	3.68E-08	1.01197	0.0106	2.62E-01	-0.0096	0.0076	2.08E-01
rs13041338	20	44283123	1.1992	0.0316	8.92E-09	1.01096	0.0105	3.01E-01	-0.01	0.0077	1.92E-01
rs13041885	20	44283477	1.1964	0.0319	1.85E-08	1.01035	0.0107	3.35E-01	-0.0097	0.0078	2.10E-01
rs6065853	20	44284023	1.1988	0.0316	9.28E-09	1.01136	0.0105	2.82E-01	-0.009	0.0076	2.37E-01
rs6104276	20	44284591	1.1983	0.0316	1.06E-08	0.98827	0.0108	2.76E-01	0.0086	0.0078	2.69E-01
rs6104278	20	44285991	1.2045	0.0351	1.13E-07	1.01207	0.0115	2.99E-01	-0.0127	0.0089	1.54E-01
rs6073835	20	44286454	1.199	0.0316	9.04E-09	1.01157	0.0106	2.78E-01	-0.0097	0.0077	2.04E-01
rs1825777	20	44287194	1.199	0.0316	9.05E-09	0.98857	0.0106	2.78E-01	0.0094	0.0077	2.22E-01
rs1586439	20	44287425	1.199	0.0316	9.03E-09	0.98896	0.0106	2.94E-01	0.0097	0.0077	2.05E-01
rs6065854	20	44288816	1.1991	0.0316	8.94E-09	0.98847	0.0106	2.73E-01	0.0094	0.0077	2.21E-01
rs6073838	20	44289156	1.2079	0.0345	4.23E-08	1.01086	0.0111	3.30E-01	-0.0108	0.0081	1.83E-01
rs6073839	20	44289328	1.2061	0.0342	4.27E-08	1.01308	0.0111	2.40E-01	-0.0103	0.0082	2.09E-01
rs6073840	20	44289370	1.2012	0.032	1.06E-08	0.99114	0.0111	4.20E-01	0.0093	0.008	2.44E-01
rs6073841	20	44290421	1.2049	0.035	1.01E-07	1.00572	0.0114	6.17E-01	-0.0099	0.0086	2.47E-01
rs6065856	20	44291022	1.1795	0.0361	4.82E-06	1.01359	0.0117	2.48E-01	-0.0107	0.0096	2.69E-01
rs6104282	20	44291055	1.1856	0.0365	3.15E-06	1.0141	0.0123	2.55E-01	-0.0055	0.0105	5.98E-01
rs6104283	20	44291410	1.1995	0.0317	9.25E-09	0.98896	0.011	3.10E-01	0.0099	0.0077	1.95E-01
rs6073842	20	44293841	1.2004	0.0317	8.04E-09	1.01116	0.0106	2.93E-01	-0.0096	0.0076	2.08E-01
rs6073843	20	44293956	1.2004	0.0317	8.04E-09	1.01126	0.0106	2.88E-01	-0.0096	0.0076	2.09E-01
rs6073844	20	44294234	1.1986	0.0318	1.18E-08	1.01136	0.0106	2.87E-01	-0.01	0.0077	1.92E-01
rs11696685	20	44295134	1.1989	0.0316	9.24E-09	0.98896	0.0105	2.93E-01	0.0098	0.0076	1.99E-01
rs6104285	20	44295416	1.1988	0.0316	9.22E-09	0.98906	0.0105	2.94E-01	0.0095	0.0076	2.15E-01
rs1487319	20	44295943	1.1975	0.0315	1.11E-08	0.98926	0.0105	3.03E-01	0.0088	0.0076	2.50E-01
chr20:44296110:D	20	44296110	1.189	0.0322	7.68E-08	0.99005	0.0107	3.49E-01			
rs6073846	20	44296323	1.1975	0.0315	1.10E-08	0.98886	0.0105	2.88E-01	0.0109	0.0076	1.52E-01
rs6094192	20	44297796	1.1975	0.0315	1.08E-08	1.01126	0.0106	2.87E-01	-0.0095	0.0076	2.13E-01
rs6073848	20	44298550	1.1975	0.0315	1.06E-08	1.01167	0.0108	2.83E-01	-0.0092	0.0076	2.31E-01
rs6073849	20	44299385	1.1974	0.0315	1.09E-08	0.98926	0.0106	3.04E-01	0.0087	0.0077	2.56E-01
chr20:44299878:D	20	44299878	1.1965	0.0316	1.38E-08	0.98995	0.0107	3.44E-01			
rs6073850	20	44301084	1.1979	0.0315	1.04E-08	1.01126	0.0106	2.90E-01	-0.0097	0.0077	2.08E-01
rs6065859	20	44302697	1.1974	0.0315	1.08E-08	1.01147	0.0105	2.81E-01	-0.0095	0.0076	2.15E-01
rs6073851	20	44306978	1.1969	0.0315	1.16E-08	1.01136	0.0106	2.83E-01	-0.0093	0.0076	2.22E-01
rs6124726	20	44307866	1.1969	0.0315	1.16E-08	1.01157	0.0105	2.78E-01	-0.0093	0.0076	2.26E-01
rs1487310	20	44308789	1.1969	0.0315	1.16E-08	0.98857	0.0106	2.77E-01	0.0092	0.0076	2.30E-01
rs6073854	20	44309605	1.1969	0.0315	1.16E-08	0.98866	0.0105	2.80E-01	0.0093	0.0076	2.25E-01
rs6073855	20	44309670	1.197	0.0315	1.16E-08	1.01157	0.0105	2.77E-01	-0.011	0.0076	1.48E-01
rs8123346	20	44309987	1.197	0.0315	1.16E-08	1.01136	0.0105	2.85E-01	-0.0092	0.0076	2.31E-01
rs1013562	20	44310744	1.1971	0.0315	1.15E-08	0.98876	0.0105	2.85E-01	0.0098	0.0078	2.08E-01
rs6065861	20	44311056	1.1969	0.0315	1.16E-08	0.98886	0.0106	2.88E-01	0.0109	0.0085	1.99E-01
rs4810465	20	44311354	1.1969	0.0315	1.16E-08	1.01147	0.0105	2.79E-01	-0.0127	0.0083	1.25E-01
rs3746596	20	44312028	1.1974	0.0315	1.11E-08	1.01136	0.0106	2.84E-01	-0.0098	0.0077	2.04E-01
rs2281211	20	44312858	1.1971	0.0315	1.16E-08	1.01157	0.0106	2.77E-01	-0.0099	0.0077	1.96E-01
rs6073856	20	44313020	1.197	0.0315	1.16E-08	1.01147	0.0105	2.79E-01	-0.0092	0.0076	2.28E-01
rs2072974	20	44313401	1.1971	0.0315	1.15E-08	1.01136	0.0106	2.84E-01	-0.0099	0.0077	1.96E-01
rs6065863	20	44316083	1.1981	0.0315	1.00E-08	1.01157	0.0106	2.81E-01	-0.0084	0.0077	2.74E-01
rs6094197	20	44316492	1.1973	0.0321	1.98E-08	1.00763	0.0109	4.87E-01	-0.0081	0.0079	3.08E-01
rs147282660	20	44317477	1.202	0.0323	1.24E-08	0.99054	0.011	3.87E-01	0.0078	0.008	3.30E-01
rs6094199	20	44321057	1.1907	0.0344	3.86E-07	0.99392	0.0117	6.00E-01	0.0111	0.0089	2.13E-01
rs146537075	20	44321432	1.1905	0.0334	1.74E-07	1.00975	0.011	3.79E-01	-0.0098	0.0081	2.27E-01
rs6073859	20	44321884	1.2022	0.0319	8.02E-09	1.01187	0.0106	2.67E-01	-0.0095	0.0078	2.19E-01
rs6104290	20	44321902	1.202	0.0319	8.29E-09	1.01227	0.0106	2.53E-01	-0.0086	0.0077	2.65E-01
rs6073860	20	44321946	1.2005	0.0321	1.27E-08	1.00985	0.0108	3.67E-01	-0.0087	0.0078	2.66E-01
rs6065865	20	44322016	1.195	0.0336	1.10E-07	1.01136	0.0111	3.10E-01	-0.0089	0.0082	2.78E-01
rs4812953	20	44322248	1.1954	0.0319	2.12E-08	1.01106	0.0107	3.01E-01	-0.0094	0.0078	2.28E-01
rs4810466	20	44322266	1.2008	0.0335	4.83E-08	1.01015	0.0109	3.58E-01	-0.0097	0.0082	2.35E-01
rs6130893	20	44322582	1.1966	0.0317	1.49E-08	0.98837	0.0106	2.67E-01	0.0092	0.0077	2.32E-01
chr20:44322603:D	20	44322603	1.1976	0.0318	1.44E-08	0.99005	0.0107	3.51E-01			
rs6130894	20	44322673	1.1966	0.0317	1.49E-08	1.01167	0.0106	2.74E-01	-0.0092	0.0077	2.32E-01
rs6104291	20	44323368	1.1966	0.0317	1.48E-08	0.98857	0.0106	2.74E-01	0.0092	0.0077	2.32E-01
rs6065866	20	44323440	1.1966	0.0317	1.48E-08	1.01167	0.0106	2.74E-01	-0.0091	0.0077	2.33E-01
rs4812955	20	44324638	1.1969	0.0317	1.43E-08	1.01136	0.0106	2.85E-01	-0.01	0.0077	1.92E-01
rs6124727	20	44325623	1.196	0.0317	1.66E-08	0.98857	0.0106	2.75E-01	0.0094	0.0077	2.20E-01

rs6073861	20	44325708	1.1967	0.0317	1.48E-08	1.01167	0.0106	2.74E-01	-0.0091	0.0077	2.36E-01
rs6104293	20	44326298	1.1967	0.0317	1.48E-08	0.98807	0.0106	2.55E-01	0.0092	0.0077	2.33E-01
rs6104294	20	44326850	1.1968	0.0317	1.47E-08	0.98857	0.0106	2.76E-01	0.0098	0.0077	2.01E-01
rs6104295	20	44326917	1.1968	0.0317	1.48E-08	1.01177	0.0106	2.70E-01	-0.0091	0.0077	2.36E-01
rs6130896	20	44327532	1.1992	0.0322	1.76E-08	0.98866	0.0106	2.81E-01	0.0093	0.0077	2.28E-01
rs6104297	20	44327621	1.2015	0.0334	3.97E-08	0.98718	0.0112	2.50E-01	0.0089	0.0081	2.67E-01
rs6104298	20	44327774	1.1989	0.0319	1.37E-08	0.98847	0.0106	2.71E-01	0.0091	0.0077	2.36E-01
rs6130897	20	44330004	1.1896	0.0336	2.31E-07	1.01136	0.0106	2.85E-01	-0.01	0.0077	1.95E-01
rs714595	20	44330591	1.1899	0.0336	2.24E-07	1.01066	0.0106	3.14E-01	-0.0104	0.0077	1.78E-01
rs6073862	20	44331705	1.1959	0.0346	2.25E-07	0.98965	0.0107	3.31E-01	0.0103	0.0078	1.89E-01
rs980984	20	44333052	0.8524	0.0357	7.45E-06	1.00995	0.0083	2.34E-01	-0.016	0.0059	6.98E-03
rs232291	20	44334394	0.8497	0.0363	7.09E-06	0.9996	0.008	9.60E-01	-0.0097	0.0061	1.13E-01
rs232290	20	44334588	0.8503	0.0362	7.58E-06	1.0006	0.008	9.37E-01	-0.0088	0.0061	1.54E-01
rs232285	20	44338769	0.8508	0.036	7.15E-06	0.9998	0.008	9.80E-01	-0.0094	0.0062	1.28E-01
rs232276	20	44343536	0.8512	0.036	7.38E-06	0.9992	0.008	9.17E-01	0.0088	0.0062	1.55E-01
rs232270	20	44346426	0.8513	0.036	7.76E-06	1.0005	0.008	9.49E-01	-0.0084	0.0063	1.80E-01
rs232268	20	44347388	0.8514	0.036	7.88E-06	1.0006	0.008	9.39E-01	-0.0086	0.0063	1.68E-01
rs232267	20	44348034	0.8516	0.036	8.04E-06	1.0004	0.008	9.64E-01	-0.0083	0.0063	1.85E-01
rs6032468	20	44349105	0.855	0.0349	7.05E-06	0.9861	0.0091	1.25E-01	0.0167	0.0063	8.57E-03
rs232263	20	44350728	0.8535	0.0359	9.97E-06	1.01116	0.009	2.18E-01	-0.0115	0.0063	6.66E-02
rs232262	20	44350931	0.8554	0.0347	6.77E-06	1.01359	0.009	1.33E-01	-0.0143	0.0062	2.16E-02
chr20:44362545:I	20	44362545	1.2142	0.0395	9.05E-07	0.99273	0.0094	4.38E-01			
chr20:44362548:I	20	44362548	1.2094	0.0393	1.32E-06	0.99094	0.0097	3.48E-01			
chr20:44362550:I	20	44362550	1.2192	0.0405	9.73E-07	0.99035	0.0097	3.19E-01			
rs6032493	20	44364750	1.1727	0.0348	4.83E-06	1.00391	0.0093	6.77E-01	-0.0145	0.0063	2.08E-02
rs2056161	20	44368763	1.1698	0.0354	9.67E-06	1.0014	0.009	8.74E-01	-0.011	0.0063	7.90E-02
rs2425746	20	44372620	1.1835	0.036	2.83E-06	0.9984	0.009	8.56E-01	0.0115	0.0063	6.79E-02
rs2425747	20	44372893	1.1858	0.0364	2.82E-06	1.0009	0.009	9.24E-01	-0.0118	0.0063	6.28E-02

Hatched pattern indicates SNPs with P value less than 5E-2 and the same direction of effect. SNP: Variant identifier, CHR: Chromosome code, BP: Base-pair coordinate, OR: odds ratio, SE: Standard error of effect estimate, P: Association test p-value
