# Supplementary material for: Genome-wide association study identifies a novel locus associated with psychological distress in the Japanese population
Source: Transl Psychiatry. 2019 Jan 31;9:52. doi: 10.1038/s41398-019-0383-z (PMC6355763; doi:10.1038/s41398-019-0383-z)
Supplement: Supplementary file 4 — Supplemental Table 4 [file 41398_2019_383_MOESM4_ESM.pdf]

Supplementary Table 4. List of locus with genome-wide significant association in the PGC2 MDD GWAS and their OR, SE, and P value observed in the present GWAS.

			MDD (PGC2 ex23andMe)			Psychological Distress (GQ)			Direction
SNP	CHR	BP	OR	SE	P	OR	SE	P	of effect
rs159963	1	8504421	0.97	0.0049	3.2E-08	0.9854	0.0405	7.16E-01	opposite
rs1432639	1	72813218	1.04	0.005	4.6E-15	0.9253	0.0591	1.89E-01	same
rs12129573	1	73768366	1.04	0.005	4.0E-12	0.9989	0.0361	9.76E-01	same
rs2389016	1	80799329	1.03	0.0053	1.0E-08	0.9785	0.0378	5.66E-01	same
rs4261101	1	90796053	0.97	0.005	1.0E-08	0.9982	0.0411	9.65E-01	opposite
rs9427672	1	197754741	0.97	0.0058	3.1E-08	1.0209	0.0394	6.00E-01	opposite
rs11682175	2	57987593	0.97	0.0048	4.7E-09	1.0159	0.03	5.98E-01	opposite
rs1226412	2	157111313	1.03	0.0059	2.4E-08	1.0072	0.0329	8.27E-01	opposite
chr3_44287760_I	3	44287760	1.03	0.0051	4.6E-08				
rs7430565	3	158107180	0.97	0.0048	2.9E-09	1.0758	0.0314	2.00E-02	opposite
rs34215985	4	42047778	0.96	0.0063	3.1E-09	0.9685	0.061	6.00E-01	same
chr5_87992715_I	5	87992715	0.97	0.005	7.9E-11				
chr5_103942055_D	5	103942055	1.03	0.0048	7.5E-12				
rs116755193	5	124251883	0.97	0.005	7.0E-09	1.0244	0.041	5.56E-01	same
rs11135349	5	164523472	0.97	0.0048	1.1E-09	0.9425	0.0376	1.15E-01	opposite
rs4869056	5	166992078	0.97	0.005	6.8E-09	1.0422	0.0332	2.13E-01	same
rs115507122	6	30737591	0.96	0.0063	3.3E-11				
rs9402472	6	99566521	1.03	0.0059	2.8E-08				
rs10950398	7	12264871	1.03	0.0049	2.6E-08	1.0012	0.0304	9.68E-01	opposite
rs12666117	7	109105611	1.03	0.0048	1.4E-08	1.077	0.0321	2.07E-02	opposite
rs1354115	9	2983774	1.03	0.0049	2.4E-08	0.927	0.0633	2.31E-01	same
rs10959913	9	11544964	1.03	0.0057	5.1E-09	0.9671	0.0391	3.92E-01	same
rs7856424	9	119733595	0.97	0.0053	8.5E-09	1.0267	0.0312	3.98E-01	same
rs7029033	9	126682068	1.05	0.0093	2.7E-08	0.8986	0.0566	5.91E-02	opposite
rs61867293	10	106563924	0.96	0.0061	7.0E-10				opposite
rs1806153	11	31850105	1.04	0.0059	1.2E-09	0.977	0.0311	4.55E-01	same
rs4074723	12	23947737	0.97	0.0049	3.1E-08	0.9446	0.0453	2.08E-01	opposite
rs4143229	13	44327799	0.95	0.0091	2.5E-08	1.0747	0.0351	4.03E-02	same
rs12552	13	53625781	1.04	0.0048	6.1E-19				
rs4904738	14	42179732	0.97	0.0049	2.6E-09	0.8853	0.0694	7.90E-02	same
rs915057	14	64686207	0.97	0.0049	7.6E-10	1.0441	0.0318	1.75E-01	opposite
chr14_75356855_I	14	75356855	1.03	0.0049	3.8E-09				
rs10149470	14	104017953	0.97	0.0049	3.1E-09	0.9817	0.0309	5.50E-01	same
rs8025231	15	37648402	0.97	0.0048	2.4E-12	0.9846	0.0314	6.22E-01	same
rs8063603	16	6310645	0.97	0.0053	6.9E-09	1.0258	0.0361	4.80E-01	same
rs7198928	16	7666402	1.03	0.005	1.0E-08	1.0008	0.0386	9.84E-01	opposite
rs7200826	16	13066833	1.03	0.0055	2.4E-08	0.9464	0.0636	3.86E-01	same
rs11643192	16	72214276	1.03	0.0049	3.4E-08	1.0485	0.0302	1.16E-01	opposite
rs17727765	17	27576962	0.95	0.0088	8.5E-09				
rs62099069	18	36883737	0.97	0.0049	1.3E-08	1.0344	0.0339	3.18E-01	opposite
rs11663393	18	50614732	1.03	0.0049	1.6E-08	1.0385	0.1215	7.56E-01	opposite
rs1833288	18	52517906	1.03	0.0054	2.6E-08	0.9522	0.0333	1.41E-01	opposite
rs12958048	18	53101598	1.03	0.0051	3.6E-11	1.0597	0.0297	5.11E-02	same
rs5758265	22	41617897	1.03	0.0054	7.6E-09	0.9576	0.0371	2.42E-01	opposite

SNP: Variant identifier, CHR: Chromosome code, BP: Base-pair coordinate, OR: odds ratio, SE: Standard error of effect estimate, P: Association test p-value
