# Supplementary material for: Genome-wide association study identifies a novel locus associated with psychological distress in the Japanese population
Source: Transl Psychiatry. 2019 Jan 31;9:52. doi: 10.1038/s41398-019-0383-z (PMC6355763; doi:10.1038/s41398-019-0383-z)
Supplement: Supplementary file 5 — Supplemental Table 5 [file 41398_2019_383_MOESM5_ESM.pdf]

Supplementary Table 5. List of locus with suggestive significant association in the GPC2 Neuroticism GWAS and their OR, SE, and P value observed in the present GWAS.

			NEUROTICISM (GPC2)			Psychological Distress (GQ)			Direction
RSID	CHR	BP	BETA	SE	P	OR	SE	P	of effect
rs35855737	3	65542856	0.0403	0.007	9.26E-09				
rs35557878	3	65573858	-0.0349	0.0069	4.69E-07				
rs2393911	6	27057079	-0.0387	0.008	1.20E-06	1.124	0.0516	2.34E-02	same
rs77337722	18	74935886	0.1746	0.036	1.24E-06	0.9882	0.0622	8.49E-01	same
rs1524970	3	65538544	-0.0306	0.0064	1.63E-06	1.0288	0.0526	5.89E-01	opposite
rs76457088	18	74936258	0.1705	0.0359	2.06E-06	0.9869	0.0621	8.32E-01	opposite
rs1811103	22	34182889	0.0388	0.0082	2.33E-06	1.0619	0.055	2.75E-01	same
rs10934447	3	118101726	0.0347	0.0074	2.53E-06	1.0007	0.0314	9.81E-01	opposite
rs4653163	1	36627445	0.1516	0.0323	2.70E-06	0.9515	0.0512	3.31E-01	opposite
rs56118546	5	164465890	-0.0388	0.0083	2.91E-06	1.0026	0.0489	9.58E-01	opposite
rs412701	22	34175587	0.04	0.0086	2.91E-06	1.0401	0.0512	4.43E-01	same
rs62188582	2	240175059	-0.1287	0.0278	3.64E-06				
rs9661830	1	23614982	0.033	0.0071	3.76E-06	1.001	0.0349	9.78E-01	same
rs72817567	5	164579303	0.0345	0.0075	3.80E-06	1.0407	0.045	3.75E-01	opposite
rs1117999	2	13755118	0.0474	0.0103	3.84E-06	1.0102	0.0308	7.41E-01	same
rs55940235	5	164581837	-0.0345	0.0075	3.87E-06	1.0404	0.045	3.80E-01	opposite
rs72817562	5	164576968	0.0345	0.0075	3.94E-06	1.0409	0.045	3.73E-01	opposite
rs72817565	5	164579250	-0.0345	0.0075	4.00E-06	1.0407	0.045	3.75E-01	opposite
rs2112175	5	164580712	-0.0344	0.0075	4.04E-06	1.0345	0.0435	4.36E-01	opposite
rs55953354	5	164581366	0.0344	0.0075	4.10E-06	1.0405	0.045	3.78E-01	opposite
rs57575916	1	23602620	-0.033	0.0072	4.15E-06	1.0011	0.0349	9.75E-01	same
rs114293326	5	164471700	0.0364	0.0079	4.22E-06	1.0011	0.0349	9.75E-01	opposite
rs649915	1	23690876	-0.0318	0.0069	4.28E-06	1.0007	0.0315	9.81E-01	same
rs3753250	1	23669770	-0.0326	0.0071	4.29E-06	1.0094	0.0351	7.89E-01	opposite
rs2039316	1	23600798	-0.0333	0.0072	4.33E-06	0.9995	0.035	9.89E-01	same
rs1421698	5	164490019	0.0355	0.0077	4.44E-06	1.013	0.0484	7.89E-01	opposite
rs17072113	5	164489105	-0.0355	0.0077	4.45E-06	1.0129	0.0485	7.92E-01	opposite
rs1421697	5	164490201	0.0355	0.0077	4.45E-06	1.0135	0.0485	7.82E-01	opposite
rs140142689	5	164488914	0.0355	0.0077	4.55E-06	1.0128	0.0485	7.92E-01	opposite
rs72808038	5	164493780	0.0353	0.0077	4.57E-06				
rs61777134	1	23691762	0.0317	0.0069	4.58E-06	1.0012	0.0315	9.71E-01	same
rs58067912	5	164475990	-0.036	0.0079	4.85E-06	1.0024	0.0495	9.62E-01	opposite
rs72808024	5	164479164	-0.0356	0.0078	4.85E-06	1.0024	0.0494	9.61E-01	opposite
rs2413200	22	34191950	0.0388	0.0085	4.90E-06	1.0629	0.0639	3.40E-01	same
rs644749	1	23687827	0.0321	0.007	4.96E-06	1.0006	0.0315	9.86E-01	same
rs7736835	5	164482610	0.0356	0.0078	4.98E-06	1.0061	0.049	9.01E-01	opposite

SNP: Variant identifier, CHR: Chromosome code, BP: Base-pair coordinate, OR: odds ratio, SE: Standard error of effect estimate, P: Association test p-value
